# Why antisense could make sense for neurodegeneration

**DOI:** 10.1007/s00415-017-8515-y

**Published:** 2017-05-17

**Authors:** T. H. Massey, N. P. Robertson

**Affiliations:** Division of Psychological Medicine and Clinical Neuroscience, Department of Neurology, Cardiff University, University Hospital of Wales, Heath Park, Cardiff, CF14 4XN UK

## Introduction

Antisense oligonucleotides (ASOs) are modified nucleic acids that alter gene expression in cells by binding target mRNA in a sequence-specific manner. ASO binding can trigger degradation or alter processing of mRNA. Potential advantages of this class of therapeutic include specificity (from base pairing), amplification of effect (one ASO can catalyse the degradation/processing of many mRNAs), and reversibility (in contrast to gene therapies acting on DNA). Many neurodegenerative diseases have either a direct genetic cause or a phenotype that is modified by genetic variation making them ideal candidates for ASO therapeutics. In practice, developing effective ASOs for neurological disease has been difficult: targeting to the appropriate part of the central nervous system (CNS) is problematic and early ASOs had short half-lives and significant inflammatory side effects. However, advances in ASO chemistry over the last 20 years have improved the pharmacodynamic, pharmacokinetic, and side-effect profiles of these drugs which are now being trialled in a range of conditions, with promising early results.

This month’s journal club examines three papers involving ASO therapeutics in neurodegeneration. The first two papers look at amyotrophic lateral sclerosis (ALS): one using cell/animal models to show the utility of ASOs and the other demonstrating the safety of intrathecal ASOs in humans. The third paper reports the exciting success of ASO therapy in treating spinal muscular atrophy (SMA).

## Poly(GP) proteins are a useful pharmacodynamic marker for *C9ORF72*-associated amyotrophic lateral sclerosis

The most common known mutation associated with ALS/frontotemporal dementia (FTD) is a G_4_C_2_ hexanucleotide DNA repeat expansion in the non-coding region of *C9ORF72*, accounting for at least 10% of all ALS/FTD (up to 40% of familial cases, at least 10% of sporadic cases). Sense and antisense transcripts of the repeat are produced in cells and can be translated in each of the three possible frames to produce six repeating dipeptides. Both RNA and protein derived from the repeat have been implicated in pathogenesis, but exact mechanisms remain unclear.

This paper investigated the utility of one of these repeating dipeptides, poly(GP), as a pharmacodynamic marker for *C9ORF72*-associated ALS. It demonstrates that poly(GP) is detectable in CSF of patients with G_4_C_2_ repeat expansion, whether symptomatic or not, and that CSF poly(GP) concentration is stable in individuals over time periods of up to 22 months. There was no significant association between poly(GP) and age at onset or progression of disease. Lymphoblastoid and induced pluripotent stem cell-derived neuronal lines from patients with *C9ORF72*-related ALS produced poly(GP) in culture, with intracellular and extracellular concentrations correlated. Treatment with ASOs against G_4_C_2_ repeats reduced both repeat-containing RNA foci and poly(GP) concentrations in cells. Finally, a G_4_C_2_ repeat-specific ASO was delivered through a single bolus injection to the right ventricle of the brains of transgenic mice expressing (G_4_C_2_)_66_ as a model of *C9ORF72*-associated ALS. Mice were injected at 4 months of age, prior to the onset of pathology, and examined at 6 months of age. Repeat-containing RNA concentrations were reduced by approximately 50% with no effect on endogenous *C9orf72* mRNA. Poly(GP) concentrations in CSF and brain were correlated and both were significantly reduced following ASO treatment. RNA foci and poly(GP)-containing inclusions were reduced in the motor cortices of ASO-treated mice.

*Comment.* In the specific context of *C9ORF72*-associated ALS/FTD, poly(GP) in CSF reflects transcription and translation of the repeat. However, poly(GP) is not a good biomarker of ALS/FTD: it was found in patients whether symptomatic or not, its presence did not correlate with disease onset or progression, and its concentration did not correlate with disease severity. As expected, ASOs against the G_4_C_2_ repeat reduced mRNA and poly(GP) concentrations in cells and mice, but the utility of poly(GP) as a pharmacodynamic marker may be clouded by its production from both sense and antisense transcripts of the repeat.

Gendron TF et al (2017) Sci Transl Med 9:eaai7866

## An antisense oligonucleotide against *SOD1* delivered intrathecally for patients with *SOD1* familial amyotrophic lateral sclerosis: a phase 1, randomised, first-in-man study

Approximately 10–20% of familial ALS (and 1–2% of sporadic ALS) is caused by a toxic gain-of-function mutation in *SOD1* leading to motor neuron loss by a poorly understood mechanism. This paper reported a first-in-man, randomised, double-blinded, placebo-controlled phase I trial of intrathecal ASO against *SOD1* mRNA (both wild type and mutant). Patients (*n* = 21) with a family history of ALS and carrying a documented heterozygous mutation in *SOD1* were enrolled into four cohorts of eight (some were re-enrolled) and treated with a single, small volume (0.25 ml) intrathecal administration of *SOD1* ASO or placebo over 11.5 h via a lumbar intraspinal catheter. Sequential cohorts received increasing doses of ASO. Although 84% of patients reported adverse events, these were mild, self-limiting, and mostly related to lumbar puncture (headache and back pain). There was no significant difference in adverse events either between treatment and placebo groups or between cohorts. No adverse reactions related to the study drug were reported. At higher ASO doses, clearance into the plasma was detected, peaking at 12 h post-administration. One patient died during the study (from ALS): post-mortem examination revealed study drug throughout the spinal cord with a gradient from lumbar to cervical.

*Comment.* This study has demonstrated that intrathecal ASO delivery is both practical and safe in patients. Side effects were mild and predictable, and related to the lumbar puncture procedure rather than the study drug. There was some clearance of drug into the plasma at higher doses which has potential safety implications, since homozygous *Sod1* knockout mice develop multiple pathologies, including hepatic cancer and motor neuropathy. Although the ASO is likely only to lower SOD1 protein levels (rather than ablating them), the long-term effects of protein-lowering need investigating, particularly as the ASO is not specific for mutant over wild-type SOD1.

Miller TM et al (2013) Lancet Neurol 12:435–442

## Treatment of infantile-onset spinal muscular atrophy with nusinersen: a phase 2, open-label, dose-escalation study

Spinal muscular atrophy (SMA) is a devastating motor neuron disease that usually presents in childhood and affects about 1 in 11,000. The most severe, infantile-onset, form of SMA (60% of all cases) presents before 6 months of age with profound muscle weakness that typically leads to early ventilator dependence and a life expectancy of less than 2 years. There have been no disease-modifying treatments available. SMA is caused by a loss-of-function mutation in the *Survival Motor Neuron 1* (*SMN1*) gene that leads to reliance on a nearby related gene, *SMN2*. *SMN2* differs only by 11 nucleotides from *SMN1* but, crucially, has a splice site enhancer mutation that means only 10–25% of its transcripts contain exon 7 and can generate full-length functional SMN protein. The remaining transcripts are degraded. The severity of SMA is related to *SMN2* copy number and hence its ability to compensate for defective *SMN1*. The ASO trialled here (‘nusinersen’) binds specifically to *SMN2* mRNA and modulates splicing, such that exon 7 is included in processed transcripts and more functional SMN2 protein is produced.

Twenty infants (between 3 weeks and 6 months of age) with SMA were treated with a loading and maintenance regimen of intrathecal nusinersen injections and followed for up to 32 months. The procedure was well tolerated and the drug safe, although all participants experienced mild–moderate adverse events. These were all deemed related to the natural history of SMA rather than drug administration. Significant improvements in developmental motor milestones, motor function, and ventilation-free survival (65 vs. 15% at 24 months of age) were recorded following treatment with nusinersen. Plasma concentrations peaked 1 h after administration and were dose dependent. Three infants died during the study from SMA: post-mortem examination showed that nusinersen was widely distributed throughout the CNS, including in deep brain structures, and also found in peripheral tissues such as liver and kidney. Analysis of thoracic spinal cord showed that nusinersen treatment was associated with dramatic increases in the amount of functional *SMN2* mRNA (21 vs. 57%) and SMN protein in motor neurons.

*Comment.* This study has provided the first direct evidence for antisense efficacy in the human CNS. Intrathecal injections of nusinersen were tolerated and safe, and delivered drug to target CNS tissues, where expected mRNA and protein effects were observed. Drug administration was associated with dramatic clinical improvements, and hence, nusinersen was taken forward to a phase III trial (ENDEAR) which was itself stopped after an interim analysis showed that 40% of patients in the treatment arm showed significant motor improvements compared to 0% in the placebo arm. Patients have now been enrolled into an ongoing open-label study (SHINE) which is running until 2020. Nusinersen has recently received FDA and EMA approval for the treatment of SMA (December 2016 and April 2017 respectively).

Finkel RS et al (2016) Lancet 388:3017–3026

## Conclusion

Intrathecal ASOs can be delivered safely in humans and induce predictable changes in gene expression in the CNS. The encouraging data relating to SMA suggest that ASOs can modify disease course in certain circumstances. However, we should be cautious when extrapolating from these results: the studies are small, the pathogenesis of SMA unusual, the follow-up time short, and the implications of widespread CNS and systemic ASO distribution unknown. In addition, ASOs targeting dominant-negative conditions are often not allele-specific, and therefore, the effects of targeting mutant and wild-type mRNAs must be carefully assessed in individual diseases.

The current trials of intrathecal ASOs in Huntington’s disease and Alzheimer’s disease, as well as ongoing studies in SMA, will provide greater understanding of these therapeutics. If results are positive, the emergence of ASOs could mark a paradigm shift in how clinicians treat genetically mediated neurodegenerative conditions.

